# Ethyl 3-hy­droxy­benzoate

**DOI:** 10.1107/S2414314626005699

**Published:** 2026-06-05

**Authors:** Alexander J. Gutzwiller, Grace E. Addison, Christine K.F. Hermann, George N. Harakas

**Affiliations:** a PO Box 6949, Radford University, Radford, Virginia 24142, USA; Universitat de les Illes Balears

**Keywords:** crystal structure, hydrogen bonding

## Abstract

The crystal of ethyl 3-hy­droxy­benzoate, C_9_H_10_O_3_, comprises two independent mol­ecules in the asymmetric unit. Each mol­ecule is approximately planar with the ethyl group directed away from the carbonyl-O atom. The planarity is reflected in the phenyl-C—C—O—C(methyl­ene) torsion angles of −179.40 (10) and 177.88 (11)°, respectively. The greatest difference between the mol­ecules is found in the relative orientations of the methyl groups as seen in the carboxyl­ate-C—O—C—C(meth­yl) torsion angles of −178.76 (11) and −171.12 (13)°, respectively. In the crystal, hydroxyl-O—H⋯O(carbon­yl) hydrogen-bonding occurs within almost planar chains along [100].

## Abstract 2

The title compound, C_9_H_10_O_3_, was synthesized through a Fischer esterification: the crystal comprises two independent mol­ecules with essentially the same planar structures.

## Graphical abstract

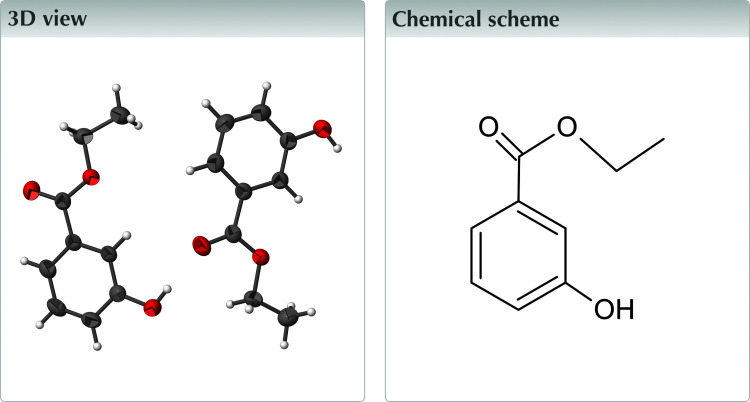

## Structure description

**Figure 1:**
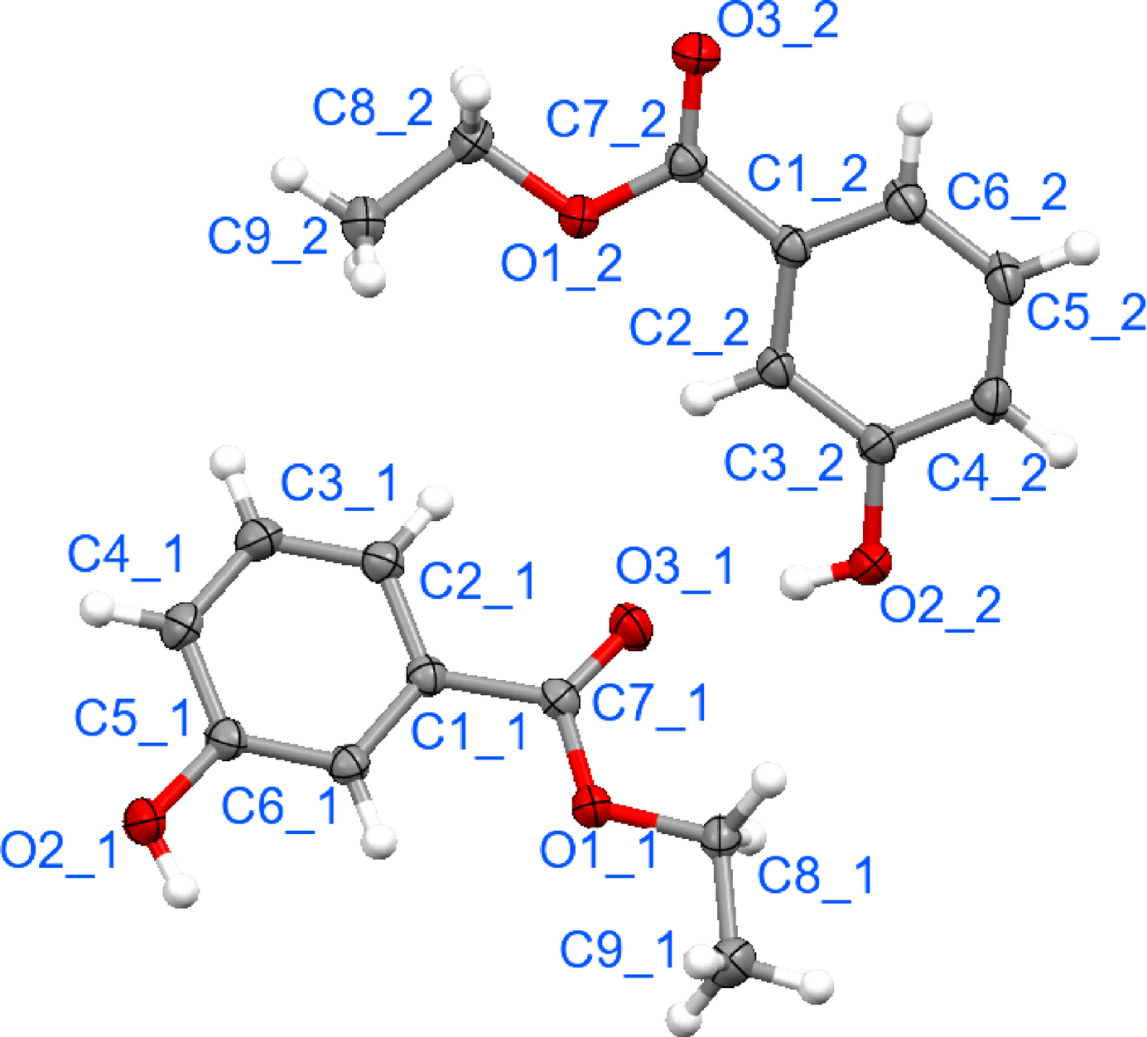
The mol­ecular structures of the two independent mol­ecules comprising the title compound showing atom labeling scheme and displacement ellipsoids at the 30% probability level.

The reaction of 3-hy­droxy­benzoic acid with ethanol yielded the title compound, ethyl 3-hy­droxy­benzoate, which has applications in organic synthesis and as a food preservative (Goretti *et al.*, 2009 ▸). The crystal structure report herein complements an undergraduate Organic Chemistry laboratory experiment (Hermann *et al.*, 2026 ▸), as detailed in Gutzwiller *et al.* (2026 ▸). The title compound crystallizes with two mol­ecules in the asymmetric unit, Fig. 1 ▸. Each independent mol­ecule is essentially planar as seen in the value of the C1_1—C7_1—O1_1—C8_1 torsion angle of −179.40 (10)°. The equivalent value for the second independent mol­ecule is 177.88 (11)°. A small deviation in the orientation of the terminal methyl groups is noted: the C7_1—O1_1—C8_1—C9_1 torsion angle is −178.76 (11)° *cf*. C7_2—O1_2—C8_2—C9_2 of −171.12 (13)°.

**Figure 2:**
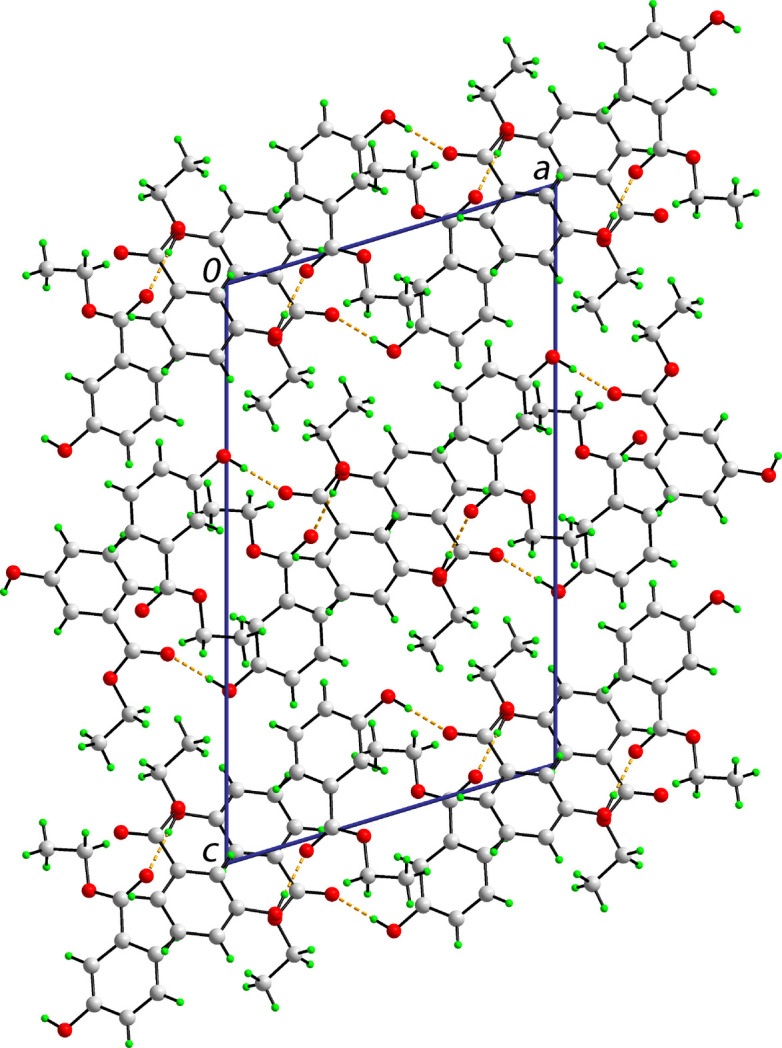
Mol­ecular packing diagram viewed in projection down the *b* axis. Hydrogen-bonding inter­actions are shown as orange dashed lines.

Hydrogen-bonding of the type hydroxyl-O—H⋯O(carbon­yl) is observed within approximately planar chains along the *a*-axis direction. The chains stack along the *b*-axis direction in an ⋯*AB*A⋯ fashion. The hydrogen-bonding parameters are given in Table 1 ▸ and a view of the unit-cell contents is shown in Fig. 2 ▸.

## Synthesis and crystallization

**Figure 3:**
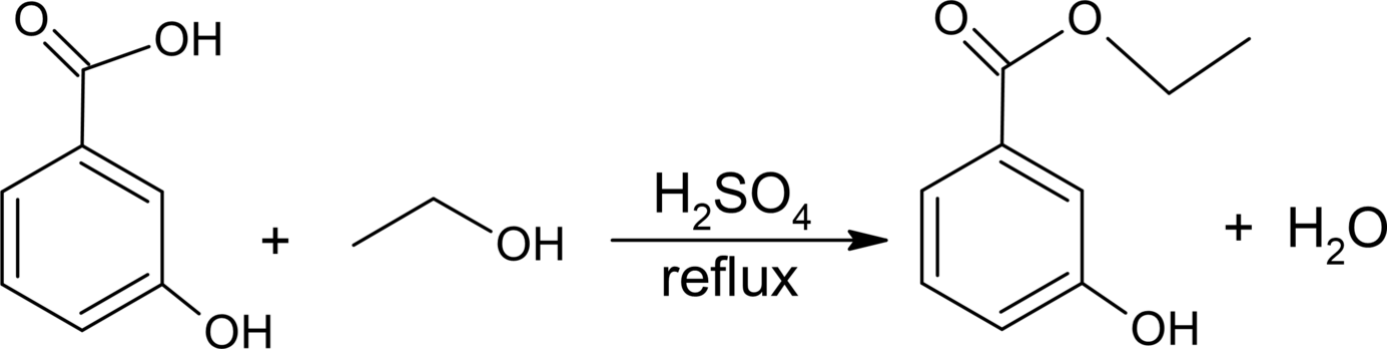
Reaction scheme for the title compound.

Referring to Fig. 3 ▸, the title compound was synthesized through a Fischer esterification. A mixture of of 3-hy­droxy­benzoic acid (1.5 g), ethanol (10 ml) and concentrated sulfuric acid (1 ml) was refluxed in a 50 ml boiling flask for 1 h. The reaction mixture was allowed to cool, then a solution of 10% sodium carbonate was added until a pH of 8 was obtained. The solution was chilled in an ice-bath until a solid product was formed. The solid was isolated by vacuum filtration.

The yield of the crude product was 0.992 g (54.3%) with a melting point of 67.2°C. X-ray quality crystals were produced by dissolving the product into methanol, followed by adding an equal volume of hexa­nes. The solvent was allowed to evaporate over several days. A single-crystal was coated with NVH oil and mounted on a MiTeGen loop then cooled to 248 K for data collection.

## Refinement

Crystal data, data collection and structure refinement details are summarized in Table 2 ▸. Owing to poor agreement, one reflection, *i.e.* 002, was omitted from the final cycles of refinement.

## Supplementary Material

Crystal structure: contains datablock(s) I. DOI: 10.1107/S2414314626005699/tk4125sup1.cif

Structure factors: contains datablock(s) I. DOI: 10.1107/S2414314626005699/tk4125Isup2.hkl

Supporting information file. DOI: 10.1107/S2414314626005699/tk4125Isup3.cml

CCDC reference: 2557954

Additional supporting information:  crystallographic information; 3D view; checkCIF report

## Figures and Tables

**Table 1:** Hydrogen-bond geometry (Å, °)

*D*—H⋯*A*	*D*—H	H⋯*A*	*D*⋯*A*	*D*—H⋯*A*
O2_1—H1_1⋯O3_2^i^	0.87 (2)	1.88 (2)	2.7495 (15)	177 (2)
O2_2—H1_2⋯O3_1	0.84 (2)	1.93 (2)	2.7617 (14)	178.1 (14)

Symmetry code: (i) 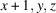.

**Table 2:** Experimental details

Crystal data	
Chemical formula	C_9_H_10_O_3_
*M*_r_	166.17
Crystal system, space group	Monoclinic, *P*2_1_/*n*
Temperature (K)	248
*a*, *b*, *c* (Å)	11.8207 (8), 7.4541 (6), 19.9477 (15)
β (°)	106.776 (2)
*V* (Å^3^)	1682.8 (2)
*Z*	8
Radiation type	Mo *K*α
μ (mm^−1^)	0.10
Crystal size (mm)	0.6 × 0.4 × 0.4

Data collection	
Diffractometer	Bruker D8
Absorption correction	Multi-scan (SADAB; Krause *et al.*, 2015 ▸)
*T*_min_, *T*_max_	0.943, 0.962
No. of measured, independent and observed [*I* > 2σ(*I*)] reflections	42216, 4174, 3152
*R*_int_	0.035
(sin θ/λ)_max_ (Å^−1^)	0.667

Refinement	
*R*[*F*^2^ > 2σ(*F*^2^)], *wR*(*F*^2^), *S*	0.042, 0.129, 1.01
No. of reflections	4174
No. of parameters	225
H-atom treatment	H atoms treated by a mixture of independent and constrained refinement
Δρ_max_, Δρ_min_ (e Å^−3^)	0.19, −0.21

Computer programs: *APEX3* and *SAINT* (Bruker, 2019 ▸), *SHELXT2018/2* (Sheldrick, 2015*a* ▸), *SHELXL2019/3* (Sheldrick, 2015*b* ▸) and *shelXle* (Hübschle *et al.*, 2011 ▸).

## full crystallographic data

### Ethyl 3-hydroxybenzoate Crystal data

**Table:**

C_9_H_10_O_3_	*F*(000) = 704
*M**_r_* = 166.17	*D*_x_ = 1.312 Mg m^−^^3^
Monoclinic, *P*2_1_/*n*	Mo *K*α radiation, λ = 0.71073 Å
*a* = 11.8207 (8) Å	Cell parameters from 9996 reflections
*b* = 7.4541 (6) Å	θ = 2.9–28.2°
*c* = 19.9477 (15) Å	µ = 0.10 mm^−^^1^
β = 106.776 (2)°	*T* = 248 K
*V* = 1682.8 (2) Å^3^	Block, colourless
*Z* = 8	0.6 × 0.4 × 0.4 mm

### Ethyl 3-hydroxybenzoate Data collection

**Table:**

Bruker D8 diffractometer	4174 independent reflections
Radiation source: sealed tube	3152 reflections with *I* > 2σ(*I*)
Flat graphite monochromator	*R*_int_ = 0.035
Detector resolution: 7.391 pixels mm^-1^	θ_max_ = 28.3°, θ_min_ = 2.3°
ω and φ scans	*h* = −15→15
Absorption correction: multi-scan (SADAB; Krause *et al.*, 2015)	*k* = −9→9
*T*_min_ = 0.943, *T*_max_ = 0.962	*l* = −26→26
42216 measured reflections	

### Ethyl 3-hydroxybenzoate Refinement

**Table:**

Refinement on *F*^2^	Primary atom site location: Intrinsic Phasing
Least-squares matrix: full	Secondary atom site location: difference Fourier map
*R*[*F*^2^ > 2σ(*F*^2^)] = 0.042	Hydrogen site location: mixed
*wR*(*F*^2^) = 0.129	H atoms treated by a mixture of independent and constrained refinement
*S* = 1.01	*w* = 1/[σ^2^(*F*_o_^2^) + (0.075*P*)^2^ + 0.2243*P*] where *P* = (*F*_o_^2^ + 2*F*_c_^2^)/3
4174 reflections	(Δ/σ)_max_ < 0.001
225 parameters	Δρ_max_ = 0.19 e Å^−^^3^
0 restraints	Δρ_min_ = −0.21 e Å^−^^3^

### Ethyl 3-hydroxybenzoate Special details

**Table:**

Geometry. All esds (except the esd in the dihedral angle between two l.s. planes) are estimated using the full covariance matrix. The cell esds are taken into account individually in the estimation of esds in distances, angles and torsion angles; correlations between esds in cell parameters are only used when they are defined by crystal symmetry. An approximate (isotropic) treatment of cell esds is used for estimating esds involving l.s. planes.

### Ethyl 3-hydroxybenzoate Fractional atomic coordinates and isotropic or equivalent isotropic displacement parameters (Å^2^)

**Table:**

	*x*	*y*	*z*	*U*_iso_*/*U*_eq_	
O1_1	0.91779 (7)	0.26186 (12)	0.52442 (4)	0.0429 (2)	
O2_1	0.99117 (9)	0.39106 (17)	0.29620 (5)	0.0585 (3)	
H1_1	1.0520 (18)	0.344 (3)	0.3269 (11)	0.088*	
O3_1	0.74342 (8)	0.38538 (14)	0.51992 (5)	0.0521 (3)	
C1_1	0.81601 (10)	0.40712 (16)	0.42054 (6)	0.0364 (3)	
C2_1	0.71478 (11)	0.49152 (18)	0.37984 (7)	0.0436 (3)	
H2_1	0.651122	0.514149	0.398019	0.052*	
C3_1	0.70881 (12)	0.5419 (2)	0.31215 (7)	0.0502 (3)	
H3_1	0.641020	0.600555	0.284399	0.060*	
C4_1	0.80135 (12)	0.5069 (2)	0.28510 (7)	0.0506 (3)	
H4_1	0.796059	0.541026	0.238929	0.061*	
C5_1	0.90218 (11)	0.42163 (18)	0.32550 (6)	0.0422 (3)	
C6_1	0.91060 (10)	0.37347 (16)	0.39395 (6)	0.0374 (3)	
H6_1	0.979587	0.318581	0.422117	0.045*	
C7_1	0.82072 (10)	0.35279 (16)	0.49288 (6)	0.0378 (3)	
C8_1	0.93052 (11)	0.20084 (19)	0.59518 (6)	0.0438 (3)	
H8A_1	0.866292	0.118519	0.595989	0.053*	
H8B_1	0.928780	0.302882	0.625844	0.053*	
C9_1	1.04619 (13)	0.1077 (2)	0.61868 (8)	0.0579 (4)	
H9A_1	1.058095	0.061953	0.665739	0.087*	
H9B_1	1.108907	0.191394	0.618440	0.087*	
H9C_1	1.047199	0.008842	0.587237	0.087*	
O1_2	0.35295 (7)	0.31978 (14)	0.37940 (4)	0.0462 (2)	
O2_2	0.64798 (8)	0.20855 (16)	0.61254 (5)	0.0547 (3)	
H1_2	0.6765 (17)	0.260 (3)	0.5839 (10)	0.082*	
O3_2	0.17933 (8)	0.23070 (15)	0.39172 (5)	0.0535 (3)	
C1_2	0.35271 (10)	0.19414 (16)	0.48736 (6)	0.0368 (3)	
C2_2	0.47221 (10)	0.23244 (16)	0.51480 (6)	0.0374 (3)	
H2_2	0.512709	0.295759	0.488075	0.045*	
C3_2	0.53107 (10)	0.17608 (17)	0.58216 (6)	0.0394 (3)	
C4_2	0.47110 (12)	0.08158 (19)	0.62121 (7)	0.0466 (3)	
H4_2	0.511232	0.043009	0.666734	0.056*	
C5_2	0.35258 (13)	0.0443 (2)	0.59319 (7)	0.0510 (3)	
H5_2	0.312368	−0.020393	0.619708	0.061*	
C6_2	0.29236 (12)	0.10108 (19)	0.52656 (7)	0.0453 (3)	
H6_2	0.211274	0.076893	0.507945	0.054*	
C7_2	0.28568 (11)	0.24977 (17)	0.41548 (6)	0.0392 (3)	
C8_2	0.29433 (12)	0.3718 (2)	0.30758 (7)	0.0522 (3)	
H8A_2	0.242762	0.475260	0.306633	0.063*	
H8B_2	0.246061	0.272506	0.282369	0.063*	
C9_2	0.38821 (14)	0.4186 (3)	0.27424 (8)	0.0666 (5)	
H9A_2	0.351992	0.451530	0.225729	0.100*	
H9B_2	0.439587	0.315993	0.276354	0.100*	
H9C_2	0.434153	0.518786	0.298951	0.100*	

### Ethyl 3-hydroxybenzoate Atomic displacement parameters (Å^2^)

**Table:**

	*U*^11^	*U*^22^	*U*^33^	*U*^12^	*U*^13^	*U*^23^
O1_1	0.0392 (5)	0.0542 (5)	0.0374 (5)	0.0024 (4)	0.0143 (4)	0.0068 (4)
O2_1	0.0482 (6)	0.0939 (8)	0.0373 (5)	0.0085 (5)	0.0187 (4)	0.0022 (5)
O3_1	0.0495 (5)	0.0623 (6)	0.0526 (6)	0.0097 (4)	0.0277 (5)	0.0096 (4)
C1_1	0.0369 (6)	0.0366 (6)	0.0361 (6)	−0.0044 (5)	0.0113 (5)	−0.0022 (5)
C2_1	0.0371 (6)	0.0491 (7)	0.0453 (7)	0.0013 (5)	0.0130 (5)	−0.0008 (6)
C3_1	0.0407 (7)	0.0624 (9)	0.0427 (7)	0.0048 (6)	0.0045 (5)	0.0028 (6)
C4_1	0.0487 (7)	0.0680 (9)	0.0328 (6)	0.0002 (7)	0.0079 (5)	0.0011 (6)
C5_1	0.0380 (6)	0.0546 (7)	0.0349 (6)	−0.0036 (5)	0.0117 (5)	−0.0058 (5)
C6_1	0.0352 (6)	0.0406 (6)	0.0359 (6)	−0.0016 (5)	0.0096 (5)	−0.0022 (5)
C7_1	0.0369 (6)	0.0370 (6)	0.0411 (6)	−0.0030 (5)	0.0139 (5)	−0.0008 (5)
C8_1	0.0457 (7)	0.0507 (7)	0.0361 (6)	−0.0046 (6)	0.0136 (5)	0.0046 (5)
C9_1	0.0536 (8)	0.0662 (9)	0.0516 (8)	0.0054 (7)	0.0116 (7)	0.0112 (7)
O1_2	0.0380 (5)	0.0658 (6)	0.0349 (5)	−0.0016 (4)	0.0105 (4)	0.0069 (4)
O2_2	0.0388 (5)	0.0853 (8)	0.0390 (5)	0.0031 (5)	0.0094 (4)	0.0112 (5)
O3_2	0.0365 (5)	0.0722 (7)	0.0497 (6)	−0.0054 (4)	0.0092 (4)	0.0034 (5)
C1_2	0.0387 (6)	0.0398 (6)	0.0349 (6)	0.0009 (5)	0.0154 (5)	−0.0035 (5)
C2_2	0.0381 (6)	0.0432 (6)	0.0347 (6)	0.0031 (5)	0.0163 (5)	0.0015 (5)
C3_2	0.0379 (6)	0.0470 (7)	0.0352 (6)	0.0062 (5)	0.0135 (5)	−0.0007 (5)
C4_2	0.0562 (8)	0.0525 (8)	0.0343 (6)	0.0053 (6)	0.0183 (6)	0.0047 (6)
C5_2	0.0595 (8)	0.0568 (8)	0.0440 (7)	−0.0099 (7)	0.0266 (6)	0.0019 (6)
C6_2	0.0441 (7)	0.0526 (8)	0.0430 (7)	−0.0088 (6)	0.0185 (5)	−0.0048 (6)
C7_2	0.0367 (6)	0.0443 (6)	0.0386 (6)	−0.0003 (5)	0.0139 (5)	−0.0034 (5)
C8_2	0.0469 (7)	0.0711 (9)	0.0354 (7)	−0.0011 (7)	0.0069 (6)	0.0061 (6)
C9_2	0.0590 (9)	0.0982 (13)	0.0464 (8)	0.0049 (9)	0.0212 (7)	0.0156 (8)

### Ethyl 3-hydroxybenzoate Geometric parameters (Å, º)

**Table:**

O1_1—C7_1	1.3248 (15)	O1_2—C7_2	1.3239 (14)
O1_1—C8_1	1.4489 (14)	O1_2—C8_2	1.4525 (15)
O2_1—C5_1	1.3620 (15)	O2_2—C3_2	1.3609 (15)
O3_1—C7_1	1.2121 (14)	O3_2—C7_2	1.2171 (15)
C1_1—C2_1	1.3878 (17)	C1_2—C6_2	1.3870 (17)
C1_1—C6_1	1.3918 (16)	C1_2—C2_2	1.3892 (17)
C1_1—C7_1	1.4842 (17)	C1_2—C7_2	1.4831 (18)
C2_1—C3_1	1.3835 (19)	C2_2—C3_2	1.3879 (17)
C3_1—C4_1	1.3773 (19)	C3_2—C4_2	1.3872 (18)
C4_1—C5_1	1.3855 (19)	C4_2—C5_2	1.378 (2)
C5_1—C6_1	1.3873 (17)	C5_2—C6_2	1.3807 (19)
C8_1—C9_1	1.484 (2)	C8_2—C9_2	1.4905 (19)
			
C7_1—O1_1—C8_1	117.09 (9)	C7_2—O1_2—C8_2	116.81 (10)
C2_1—C1_1—C6_1	120.70 (11)	C6_2—C1_2—C2_2	120.70 (12)
C2_1—C1_1—C7_1	118.46 (11)	C6_2—C1_2—C7_2	117.82 (11)
C6_1—C1_1—C7_1	120.83 (11)	C2_2—C1_2—C7_2	121.48 (11)
C3_1—C2_1—C1_1	119.11 (11)	C3_2—C2_2—C1_2	119.21 (11)
C4_1—C3_1—C2_1	120.56 (13)	O2_2—C3_2—C4_2	117.27 (11)
C3_1—C4_1—C5_1	120.41 (12)	O2_2—C3_2—C2_2	122.56 (11)
O2_1—C5_1—C4_1	117.93 (11)	C4_2—C3_2—C2_2	120.18 (12)
O2_1—C5_1—C6_1	122.29 (12)	C5_2—C4_2—C3_2	119.93 (12)
C4_1—C5_1—C6_1	119.77 (12)	C4_2—C5_2—C6_2	120.66 (12)
C5_1—C6_1—C1_1	119.42 (11)	C5_2—C6_2—C1_2	119.33 (12)
O3_1—C7_1—O1_1	123.51 (11)	O3_2—C7_2—O1_2	123.32 (12)
O3_1—C7_1—C1_1	123.80 (11)	O3_2—C7_2—C1_2	123.18 (11)
O1_1—C7_1—C1_1	112.68 (10)	O1_2—C7_2—C1_2	113.49 (10)
O1_1—C8_1—C9_1	106.37 (11)	O1_2—C8_2—C9_2	107.34 (11)
			
C6_1—C1_1—C2_1—C3_1	0.08 (19)	C6_2—C1_2—C2_2—C3_2	0.13 (18)
C7_1—C1_1—C2_1—C3_1	−179.59 (12)	C7_2—C1_2—C2_2—C3_2	−179.47 (11)
C1_1—C2_1—C3_1—C4_1	0.8 (2)	C1_2—C2_2—C3_2—O2_2	179.98 (11)
C2_1—C3_1—C4_1—C5_1	−0.4 (2)	C1_2—C2_2—C3_2—C4_2	0.45 (18)
C3_1—C4_1—C5_1—O2_1	−179.71 (13)	O2_2—C3_2—C4_2—C5_2	−179.87 (12)
C3_1—C4_1—C5_1—C6_1	−0.9 (2)	C2_2—C3_2—C4_2—C5_2	−0.3 (2)
O2_1—C5_1—C6_1—C1_1	−179.45 (12)	C3_2—C4_2—C5_2—C6_2	−0.4 (2)
C4_1—C5_1—C6_1—C1_1	1.76 (19)	C4_2—C5_2—C6_2—C1_2	1.0 (2)
C2_1—C1_1—C6_1—C5_1	−1.37 (18)	C2_2—C1_2—C6_2—C5_2	−0.83 (19)
C7_1—C1_1—C6_1—C5_1	178.29 (11)	C7_2—C1_2—C6_2—C5_2	178.79 (12)
C8_1—O1_1—C7_1—O3_1	−0.51 (18)	C8_2—O1_2—C7_2—O3_2	−1.23 (19)
C8_1—O1_1—C7_1—C1_1	−179.40 (10)	C8_2—O1_2—C7_2—C1_2	177.88 (11)
C2_1—C1_1—C7_1—O3_1	−3.67 (19)	C6_2—C1_2—C7_2—O3_2	6.19 (19)
C6_1—C1_1—C7_1—O3_1	176.66 (12)	C2_2—C1_2—C7_2—O3_2	−174.20 (12)
C2_1—C1_1—C7_1—O1_1	175.22 (11)	C6_2—C1_2—C7_2—O1_2	−172.92 (11)
C6_1—C1_1—C7_1—O1_1	−4.45 (16)	C2_2—C1_2—C7_2—O1_2	6.69 (17)
C7_1—O1_1—C8_1—C9_1	−178.76 (11)	C7_2—O1_2—C8_2—C9_2	−171.12 (13)

### Ethyl 3-hydroxybenzoate Hydrogen-bond geometry (Å, º)

**Table:**

*D*—H···*A*	*D*—H	H···*A*	*D*···*A*	*D*—H···*A*
O2_1—H1_1···O3_2^i^	0.87 (2)	1.88 (2)	2.7495 (15)	177 (2)
O2_2—H1_2···O3_1	0.84 (2)	1.93 (2)	2.7617 (14)	178.1 (14)

Symmetry code: (i) *x*+1, *y*, *z*.
